# Molecular characterization of a new member of the lariat capping twin-ribozyme introns

**DOI:** 10.1186/1759-8753-5-25

**Published:** 2014-09-15

**Authors:** Yunjia Tang, Henrik Nielsen, Benoît Masquida, Paul P Gardner, Steinar D Johansen

**Affiliations:** 1 RNA and Molecular Pathology group, Department of Medical Biology, Faculty of Health Sciences, UiT - The Arctic University of Norway, MH-building Breivika, N-9037 Tromsø, Norway; 2 Department of Cellular and Molecular Medicine, The Panum Institute, University of Copenhagen, Copenhagen, Denmark; 3 Génétique Moléculaire, Génomique, Microbiologie, IPCB, Université de Strasbourg, CNRS, Strasbourg, France; 4 School of Biological Sciences, University of Canterbury, Christchurch, New Zealand

**Keywords:** *Allovahlkampfia*, circular RNA, Heterolobosea, intron splicing, LC ribozyme, *Naegleria*, RNA structure

## Abstract

**Background:**

Twin-ribozyme introns represent a complex class of mobile group I introns that harbour a lariat capping (LC) ribozyme and a homing endonuclease gene embedded in a conventional self-splicing group I ribozyme (GIR2). Twin-ribozyme introns have so far been confined to nucleolar DNA in *Naegleria* amoeboflagellates and the myxomycete *Didymium iridis*.

**Results:**

We characterize structural organization, catalytic properties and molecular evolution of a new twin-ribozyme intron in *Allovahlkampfia* (Heterolobosea). The intron contains two ribozyme domains with different functions in ribosomal RNA splicing and homing endonuclease mRNA maturation. We found *Allovahlkampfia* GIR2 to be a typical group IC1 splicing ribozyme responsible for addition of the exogenous guanosine cofactor (exoG), exon ligation and circularization of intron RNA. The *Allovahlkampfia* LC ribozyme, by contrast, represents an efficient self-cleaving ribozyme that generates a small 2′,5′ lariat cap at the 5′ end of the homing endonuclease mRNA, and thus contributes to intron mobility.

**Conclusions:**

The discovery of a twin-ribozyme intron in a member of Heterolobosea expands the distribution pattern of LC ribozymes. We identify a putative regulatory RNA element (AP2.1) in the *Allovahlkampfia* LC ribozyme that involves homing endonuclease mRNA coding sequences as an important structural component.

## Background

Group I introns are commonly found as insertion elements in the nucleolar ribosomal DNA (rDNA) of eukaryotic microorganisms, where they interrupt highly conserved sites, both in small subunit (SSU) and large subunit (LSU) ribosomal RNA (rRNA) genes [1]. Group I introns are inherited vertically or horizontally, and are often referred to as selfish genetic elements. Most nucleolar group I introns are ribozymes (catalytic RNAs) that perform self-splicing as naked RNA *in vitro* by a common mechanism based on two coupled transesterification reactions [2,3]. Splicing is initiated by nucleophilic attack at the 5′ splice site (SS) by the 3′OH group of an exogenous guanosine cofactor (exoG). This exoG becomes covalently bound at the 5′ end of the intron RNA, and subsequently, in a second reaction, the free 3′OH group of the 5′ exon attacks the phosphodiester bond at the 3′SS. This process results in the release of the intron RNA as a linear molecule and the accurate ligation of the flanking exons. Additional and alternative transesterification reactions occur during intron RNA processing and result in full-length or truncated circular forms of the intron [1,4,5].

A well-defined and highly conserved RNA core-structure is responsible for the catalysis. A group I ribozyme core is organized into three helical stacks, named the catalytic domain (P3 and P7, proximal P8 and P9), the substrate domain (P1 and proximal P2), and the scaffold domain (P4, P5 and P6) [1,6]. About 5% to 10% of all known nucleolar group I introns harbour homing endonuclease gene (HEG) insertions. Homing endonuclease genes, which encode homing endonucleases (HEs) involved in intron mobility [7,8], are located as large insertions within the peripheral parts of the helices P1, P2, P6, P8 or P9. Here, unconventional expression strategies are utilized for these protein-coding genes embedded in nucleolar rDNA [9].

The most complex nucleolar group I introns known are the twin-ribozyme introns [3,10]. These introns consist of a regular group I splicing ribozyme (GIR2) with an insertion in P2 or P6 that contains a HEG as well as a lariat capping ribozyme (LC ribozyme). Whereas GIR2 is responsible for intron forward and reverse splicing and intron RNA circularization [5,8,11-13], the LC ribozyme performs the 2′,5′ branching reaction at an internal intron site and is directly involved in 5′ capping of the HE mRNA, a prerequisite for intron mobility [14-17].

Two natural variants of twin-ribozyme introns have been reported: the myxomycete *Didymium iridis* intron Dir.S956-1 [11,18], and introns (Nae.S516) in various species and isolates of the *Naegleria* amoeboflagellate [12,19]. Whereas both intron variants self-splice as naked RNA, express functional HEs and have a similar overall structural composition, a number of differences in distribution, inheritance and structural organization have been reported [10]. The *Didymium* LC ribozyme (DirLC; formerly DiGIR1) has been investigated in more detail and recent reports include studies of the catalytic reaction, RNA conformational changes, RNA:RNA interactions, 3D models and high-resolution X-ray crystal structures [10,16,20-22]. Similarly, structural and functional properties of *Naegleria* LC ribozymes (NaeLC; formerly NaGIR1) have been studied [12,15,17,19,23]. Importantly, both DirLC and NaeLC perform the branching reaction but contain different sequence motifs and structures in the flanking regulatory domains [17].

Here we report an expansion of the distribution pattern of twin-ribozyme introns. *Allovahlkampfia* sp., a species that belongs to the Heterolobosea amoeba, contains a twin-ribozyme intron (Asp.S516). Asp.S516 resembles the *Naegleria* introns in sequence, organization and insertion site. We have characterized the catalytic properties of the Asp.S516 RNA transcript *in vitro. Allovahlkampfia* GIR2 performs both self-splicing and complex intron RNA circle formations. We also show that the *Allovahlkampfia* LC ribozyme (AspLC) is an efficient branching ribozyme that generates a small 2′,5′ lariat cap at the 5′ end of the HE mRNA. Finally, by comparative sequence analysis we find supporting evidence that a putative regulatory RNA element known from the DirLC crystal structure is conserved in AspLC, but in a surprising context.

## Results

### *Allovahlkampfia* contains an optional group I intron at position S516 in the SSU rRNA gene

A BLAST search in the National Center for Biotechnology Information (NCBI) database using the *Naegleria jamiesoni* twin-ribozyme group I intron [12] as a query sequence identified several homologous introns in various *Naegleria* isolates [3,19], as well as a significant hit towards a nuclear SSU rDNA intron of Heterolobosea sp. BA (accession number DQ388519 [24]). The Heterolobosea sequence corresponds to a 1310 bp group I intron at position S516. Position 516 in the SSU rRNA gene is a common group I intron insertion site among eukaryotic microorganisms [19,25] and hosts the *Naegleria* twin-ribozyme intron.

Phylogenetic analysis has previously identified the Heterolobosea sp. BA as a very close relative to *Allovahlkampfia spelaea*, owing to almost identical SSU rRNA gene sequences, and as a sister species to the Acrasid slime moulds, but clearly distinct from the free-living *Naegleria* amoeboflagellates [26,27]. Thus, we renamed the Heterolobosea sp. BA isolate *Allovahlkampfia* sp. and its corresponding intron Asp.S516, according to the current nomenclature of group I introns in rDNA [28]. Asp.S516 appears optional in *Allovahlkampfia* sp. since only the BA isolate harbours an intron at the S516 site [27].

### Asp.S516 is organized as a twin-ribozyme intron composed of two distinct ribozyme domains and a homing endonuclease gene

**Figure 1:**
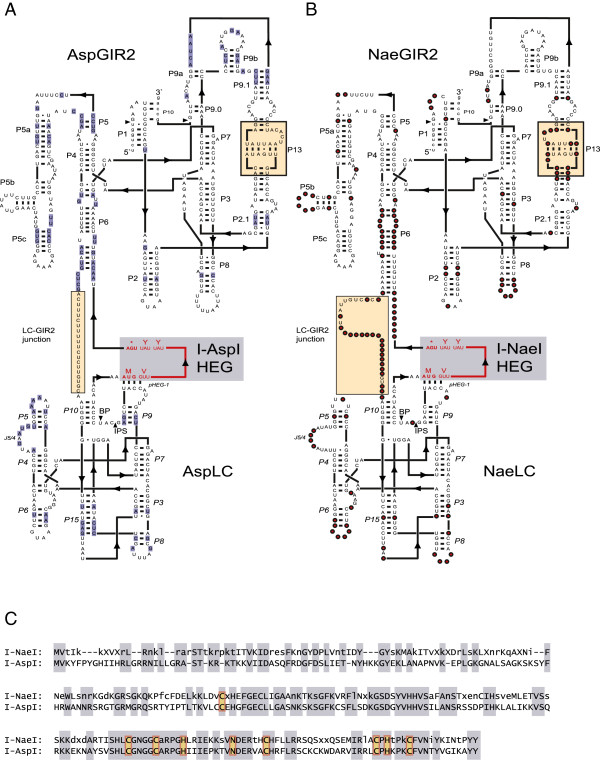
**Schematic presentation of the twin-ribozyme introns in *Allovahlkampfia *sp. (Asp.S516) and *Naegleria *(Nae.S516). (A)** Secondary structure diagram of Asp.S516 folded according to previously reported models [19] with some modifications. Asp.S516 contains two ribozyme domains, AspLC and AspGIR2, and a homing endonuclease gene (I-*Asp*I HEG). Paired RNA segments are denoted *P3* to *P15* in AspLC and P1 to P13 in AspGIR2. Nucleotide positions invariant among the *Naegleria* isolates but different in *Allovahlkampfia* are indicated in blue. Regions in Asp.S516 that deviate from Nae.S516 are boxed (LC-GIR2 junction; P13). ** (B)** Consensus secondary structure diagram of Nae.S516 based on 13 *Naegleria* introns [19]. Red circles represent variable positions based on at least one deviating *Naegleria* intron. Invariant positions are presented as uppercase letters. **(C)** Alignment of the His-Cys homing endonucleases encoded by Asp.S516 and Nae.S516. Identical residues are boxed, and deletions are indicated by dashes. Functionally important C, H and N residues involved in zinc binding and catalysis are indicated (yellow boxes). The I-*Nae*I sequence represents a consensus of nine *Naegleria* HEs (see Additional file 2: Figure S2). Uppercase letter, invariant amino acid position; lowercase letter, conserved amino acid position in at least five of nine sequences; X, non-conserved amino acid position.

A secondary structure diagram of Asp.S516 is presented in Figure 1A and compared with the consensus structure diagram of the *Naegleria* Nae.S516 twin-ribozyme intron (Figure 1B) [19]. Asp.S516 and Nae.S516 are similar in the overall structural features, and consist of three domains representing distinct structures and functions. Here, the GIR2 splicing ribozyme contains a large insertion sequence in P6 that harbours the HEG and the LC ribozyme. We previously reported that Nae.S516 was inherited vertically during evolution, and that NaeLC and HEG are evolutionary linked [19]. To further investigate the evolutionary relationship between Asp.S516 and Nae.S516, we performed phylogenetic tree analysis and found an apparent basal position of Asp.S516 compared with the *Naegleria* twin-ribozyme introns (Additional file 1: Figure S1).

The Asp.S516 HEG corresponds to a 252 amino acid His-Cys box HE with only limited sequence similarity to the 245 amino acid *Naegleria* HEs (Additional file 2: Figure S2). Whereas the amino acid identity among different *Naegleria* HEs varies from 81% to 100% [19], the *Allovahlkampfia* HE (hereafter named I-*Asp*I) contains only 46% identical positions, as compared with the consensus *Naegleria* HE (Figure 1C). Four of the *Naegleria* HEs have previously been tested and shown to cleave the intron-less rDNA allele [15,29,30]. Since I-*Asp*I shares all residues known to be essential for HE active site definition, catalysis and zinc coordination (Additional file 2: Figure S2) [19], it is likely that I-*Asp*I represents a functional HE.

AspGIR2 represents a typical group IC1 splicing ribozyme similar in sequence and structure to the *Naegleria* S516 GIR2 (Figure 1A,B) and with structural resemblance to the well-studied *Tetrahymena* intron [31]. Consistent with a more distant relationship of AspGIR2 to NaeGIR2, we detected 73 nucleotide substitutions present in *Allovahlkampfia* at invariant positions among *Naegleria* isolates (Figure 1A). The L5b-P6 GNRA-tetraloop interaction proposed to be a more recently derived structural feature in *Naegleria*[19] is missing in AspGIR2, and represents further support of a more basal position. Indels in AspGIR2 compared with NaeGIR2 are practically lacking, except for one notable example in the sequences flanking the P13 kissing-loop structure. Whereas NaeGIR2 has a 3-nt insertion, 5′ of P13 (P9 domain), AspGIR2 has a corresponding 5-nt insertion at the 3′ side (boxed in Figure 1A,B).

**Figure 2:**
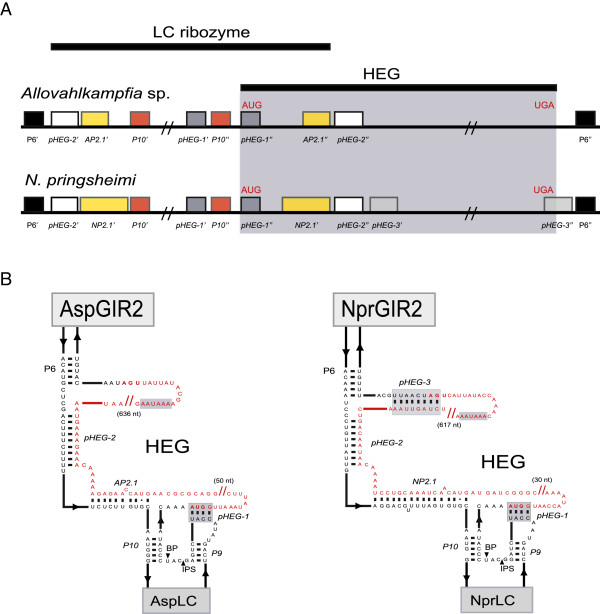
**Proposed base-pairing interactions between the LC ribozyme and HEG. (A)** Schematic box presentation of pHEG-1, pHEG-2 and AP2.1 in *Allovahlkampfia* and pHEG-1, pHEG-2, pHEG-3, and NP2.1 in *N. pringsheimi* twin-ribozyme introns, respectively. P6 is from the GIR2 ribozyme. 5′ (n′) and 3′ (n″) sequence boxes are indicated, as well as the start (AUG) and stop (UGA) codons of the HEG. **(B)** Secondary structure diagram of the LC-GIR2 junctions in Asp.S516 and Npr.S516. Corresponding HEGs (red letters) are indicated by start codon (AUG), polyA signal (AAUAAA), and stop codon (UGA). pHEG-1, pHEG-2 and P2.1 are present in both introns and indicate base pairings between the LC ribozyme and HEG. AspGIR2 and NprGIR2, group I splicing ribozymes; AspLC and NprLC, lariat capping ribozymes; BP, branch point nucleotide; IPS, internal processing site.

The AspLC ribozyme represents the *Naegleria*-type of LC ribozymes and contains the hallmark P3/P15 pseudoknot found in all LC ribozymes described to date [11,12,32]. AspLC contains 33 positions that deviate from the corresponding invariant positions in NaeLC ribozymes, but all are consistent with the secondary structure model (Figure 1A). The changes are mostly located in loop regions or found as compensatory changes in stems, including four compensatory base-pair changes in P15. In addition, unique nucleotide variants in P5 and J5/4 regions not seen among the *Naegleria* LC ribozymes are observed. Of most interest is the LC-GIR2 junction, which is only 20 nt in Asp.S516 but 31 nt in Nae.S516 (Figure 1A,B). This region is supposed to carry regulatory sequences responsible for conformation switching in LC ribozymes [10]. A closer inspection of LC-GIR2 junction sequences in Asp.S516 and all *Naegleria* S516 twin-ribozyme introns revealed highly conserved base-pairing features (named pHEG-2 and P2.1) that involve the 5′ part of the HE mRNA (Figure 2A). This structure is further supported by compensatory base pairings between the *Allovahlkampfia* and *Naegleria* introns, and among the different *Naegleria* intron species. Furthermore, the larger size of the LC-GIR2 junction in Nae.S516 compared with Asp.S516 results from the presence of a paired extension in NP2.1 and by pHEG-3 (Figure 2B).

### AspGIR2 performs intron self-splicing and intron RNA circularization

**Figure 3:**
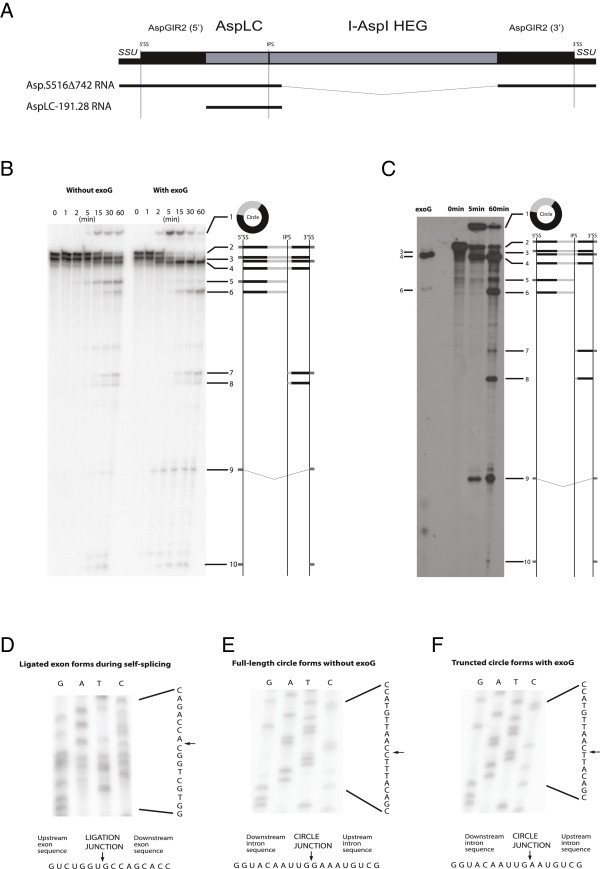
**RNA processing activity by AspGIR2. (A)** Schematic organization of Asp.S516 intron and corresponding splicing RNA construct (Asp.S516Δ742 RNA) and lariat capping RNA construct (AspLC-191.28 RNA). SSU; small subunit ribosomal RNA exons. **(B)** Time-course gel analysis of Asp.S516 self-splicing products. *In-vitro* transcribed ^32^P-labelled RNA from Asp.S516Δ742 was incubated at self-splicing with and without exoG. RNA species generated are indicated to the right. RNA#1, circular intron; RNA#2, precursor transcript; RNA#3, 5′SS or 3′SS processed precursor transcript; RNA#4, excised linear intron; RNA#5, IPS processed 5′ precursor; RNA#6, IPS processed 5′ intron; RNA#7, IPS processed 3′ precursor; RNA#8, IPS processed 3′ intron; RNA#9, ligated exon; RNA#10, free 5′ or 3′ exons. Exons, AspLC and AspGIR2 are indicated as dark grey, black and light grey boxes, respectively. **(C)** ExoG-labelling of Asp.S516Δ742 RNA compared with uniformly labelled time-course experiment of the same RNA. RNA#4 (excised linear intron) is the main labelled RNA species. **(D)** Ligation junction sequence of ligated exons generated in self-splicing reaction with exoG. Sequences were determined from RT-PCR generated products of ligated exons (RNA#9, Figure 3B) using primer combinations C716/C736 in amplification and C736 in sequencing reactions. DNA sequence reads from the reactions (right) and is complementary to that of the RNA sequences presented. Arrow marks intron insertion site or ligated exon site. **(E)** Full-length circle-junction sequences of Asp.S516 generated in self-splicing reaction without exoG. Sequences were determined from RT-PCR generated products of spliced Asp.S516Δ742 RNA using primer combination C734/C735 in amplification and C734 in sequencing reactions. The signal one nucleotide above the branch point is due to a capping-independent primer extension stop. Note that the sequencing ladder is generated from the opposite strand. **(F)** Dominating circle junction generated with exoG in the self-splicing reaction. The sequence corresponds to truncated circles lacking the first two nucleotides of the intron.

Asp.S516 DNA including parts of the flanking exons, but lacking most of the HEG was synthesized *in vitro* and cloned into a plasmid vector (Figure 3A). Corresponding *in vitro* transcribed RNA (Asp.S516Δ742) was made from a PCR product generated by amplification using an upstream exon primer containing the T7 promoter (C716) and a downstream exon primer (C717; Additional file 3: Table S1), and subjected to self-splicing, exoG-labelling, or intron circularization conditions. Figure 3B presents a time-course gel analysis of the RNA processing and self-splicing products generated after incubation at self-splicing conditions with or without exoG. Several important features are noted: (1) The ligated exon was stimulated in the presence of exoG (RNA#9); (2) the excised full-length intron was accumulated more efficiently at splicing conditions with exoG than without (RNA#4); (3) circular intron RNA species were generated both with and without exoG, but with different patterns during the time-course reaction (RNA#1); and (4) internal intron processing independent of exoG was observed (RNA#7 and RNA#8).

The RNA corresponding to ligated exons (RNA#9) was further assessed by RT-PCR amplification and sequencing following elution from the gel. As expected, the unique sequence resulting from exons ligation was identified (Figure 3D). In group I intron self-splicing, exoG becomes covalently attached to the 5′ end of the excised intron RNA. This reaction can be monitored in a radioactive exoG-labelling experiment. In the exoG-labelling experiment of the Asp.S516Δ742 RNA (Figure 3C), one of the RNA species (RNA#4) became strongly labelled during splicing, and corresponds to the excised linear intron. We infer that Asp.S516 performs exoG-dependent self-splicing catalyzed by the AspGIR2 ribozyme.

Circularization of intron RNA during self-splicing and subsequent processing is a common feature among group I introns [5,33], and three distinct pathways of generating intron RNA circles have been described and reviewed [3]. Each pathway has a unique sequence hallmark at the circularization junction and can be easily distinguished by RT-PCR sequencing. To evaluate circle identities, RNA#1 (Figure 3B) was eluted from the gel and subjected to RT-PCR sequencing. Full-length intron circle RNA was generated at reaction conditions lacking exoG (Figure 3E), an observation consistent with the splicing-independent circularization pathway initiated by 3′ SS hydrolysis of precursors [5]. A different result was observed from the reaction containing exoG. Here, a truncated circle lacking the first two nucleotides at the 5′ end of the intron was found (Figure 3F). Truncated circles are generated from excised intron RNAs [34]. A third circularization pathway incorporates exoG in the circular RNA and is also generated from the excised introns during splicing [35]. However, no support of exoG-containing intron circles was obtained since the exoG-labelling experiment did not detect any labelled circular RNAs (Figure 3C) and the RT-PCR sequencing approach did not reveal a circle junction with an additional G nucleotide. We conclude that Asp.S516 performs two types of intron circularization reactions, one dependent and one independent of exoG.

### AspLC performs 5′ lariat capping at the intron homing endonuclease mRNA

**Figure 4:**
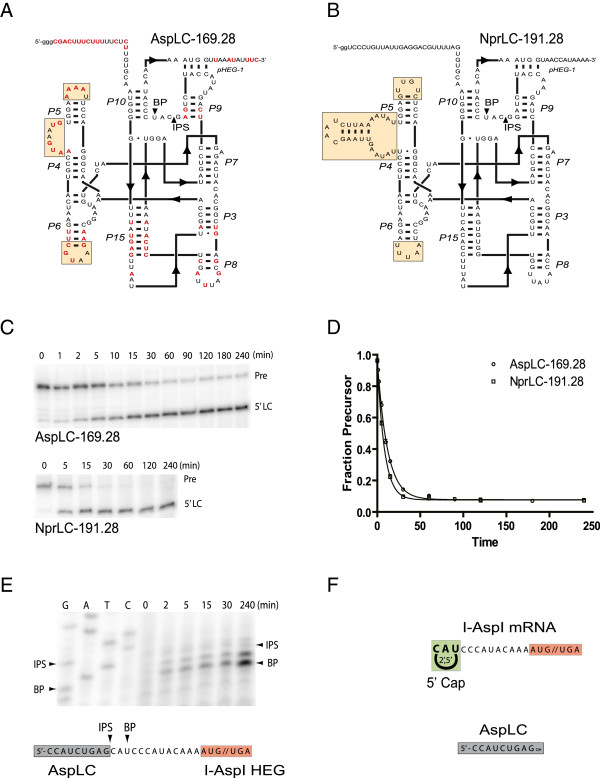
**Structure and cleavage activity of AspLC.** Secondary structure diagrams of the active forms of **(A)***Allovahlkampfia* AspLC-169.28 and **(B)***Naegleria pringsheimi* NprLC-191.28. Substitutions in AspLC compared with NprLC are indicated in red. Helical indels are boxed. **(C)** Time-course cleavage analysis of AspLC-169.28 and NprLC-191.28 transcripts separated on 5% urea polyacrylamide gels. Pre, LC precursor RNA; 5′LC, 5′ cleavage product containing the LC ribozyme sequence. **(D)** Kinetic cleavage analysis of AspLC-169.28 and NprLC-191.28 transcripts presented as fraction of uncleaved precursor versus time. **(E)** Primer extension analysis of the same sample of AspLC-169.28 analyzed in C and D, but tracking the 3′ cleavage product. BP, branch point nucleotide; IPS, internal processing site. A sequencing ladder made by sequencing of the AspLC-169.28 plasmid construct with the same primer (C720) as used for primer extension is shown. Interpretations of IPS and branch point are presented below the primer extension analysis. **(F)** Schematic presentation of the lariat capped 3′ cleavage product (I-*Asp*I mRNA) and released 5′ cleavage product (AspLC ribozyme sequence).

The observed exoG-independent cleavage at the internal processing site (IPS; Figure 3C) was likely to be catalyzed by the AspLC ribozyme. Based on recent structural and functional characterization of the *N. pringsheimi* LC ribozyme (NprLC) [17], we designed an AspLC construct with 169 nt upstream from and 28 nt downstream from the IPS (AspLC-169.28) for kinetic cleavage analysis (Figure 4A). This construct was made from a template generated by amplification of the Asp.S516Δ742 intron using the upstream primer C718 containing a T7 promoter and a downstream primer (C719; Additional file 3: Table S1). The complete AspLC-169.28 and NprLC-191.28 ribozymes (Figure 4A,B) were subjected to LC conditions incubated at different time points from 0 to 240 min, and subsequently separated by denaturing polyacrylamide electrophoresis (Figure 4C). AspLC was found to be a very efficient self-cleaving ribozyme (k_obs_ 0.08; endpoint 0.08), almost as active as the corresponding NprLC (Npr191.28; k_obs_ 0.12; endpoint 0.08) (Figure 4D).

*Naegleria* LC ribozymes generate a 3-nt lariat cap structure at the IPS that constitutes the 5′ end of the downstream HE mRNA [17]. The lariat cap structure can be detected experimentally by several approaches, including a primer extension assay [16,17]. Primer extension generates a stop at the branch point nucleotide when the cap is present, or at the IPS nucleotide when cleaved by hydrolysis. Figure 4E presents a primer extension experiment of the cleavage product after AspLC catalysis at six different time points. A strong signal was observed at the branch point nucleotide located only 10 nt upstream from the AUG start codon (Figure 4E). This result is consistent with a lariat cap structure present at the majority of 5′ ends. We interpret that AspLC is an active LC ribozyme able to generate a 3-nt 2′,5′ cap at the 5′ end of the downstream HE mRNA (Figure 4F).

## Discussion

### Expanded distribution of twin-ribozyme group I introns

We showed that Asp.S516, present in the Heterolobosea amoeba species *Allovahlkampfia* sp., is a twin-ribozyme intron. This finding expands the distribution pattern of mobile twin-ribozyme introns beyond *Naegleria* amoeboflagellate and *Didymium* myxomycete genera. Asp.S516 is related to the *Naegleria* introns and consists of two ribozymes with distinct functions in splicing and mRNA maturation. AspGIR2 represents a typical group IC1 ribozyme that catalyzes exoG-dependent intron excision and exon ligation, as well as the formation of full-length and truncated intron RNA circles. The AspLC ribozyme, on the other hand, is a new member of the LC ribozymes, and is responsible for forming a 5′ cap at the intron HE mRNA.

In Myxomycetes, only one isolate (*D. iridis* Panama 2) has been reported to harbour a mobile twin-ribozyme intron [8,11,18], despite extensive searches for nucleolar group I introns in Myxomycetes [36]. *Naegleria* twin-ribozyme introns appear much more frequent in nature, and 29 out of 70 isolates investigated harbour the Nae.S516 intron [10,19]. Nae.S516 is strictly vertically inherited, a feature that includes both ribozyme domains (NaeLC and NaeGIR2) and the HEG [19]. Furthermore, structural characterizations suggested that the intron was gained as a pre-organized genetic element early in the evolution of the *Naegleria* genus. Our recent finding of a similar twin-ribozyme intron in the more distantly related *Allovahlkampfia* indicates an even earlier occurrence.

### Role of circular intron RNAs

A biological role of group I introns beyond splicing has been debated and recently reviewed [1,3,37]. The fact that Asp.S516 and Nae.S516 are optional in *Allovahlkampfia* and *Naegleria* genera supports the notion of group I introns as selfish genetic elements, but a more complex picture appears when considering RNA processing data. Intron splicing catalyzed by AspGIR2 results in ligation of the essential host SSU rRNA. Thus, the host is dependent on a functional ribozyme in order to survive. Furthermore, the excised intron RNA has the potential of performing reverse splicing into cognate or non-cognate RNA sequence sites [13,38], and the generation of truncated intron circles from excised group I introns has been suggested to reduce or eliminate the reverse splicing reaction [5]. Interestingly, very similar truncated circles are generated both from Asp.S516 and Nae.S516 during self-splicing in the presence of exoG (Figure 3C) [12], and this suggests that truncated circles represent a conserved RNA processing feature.

The full-length intron circles, however, are generated from a different pathway and initiated by hydrolytic cleavage at the 3′SS of precursor RNAs [5]. AspGIR2 catalyzes the formation of full-length intron circles in the absence of exoG, and subsequently generates non-ligated and presumably non-functional SSU rRNAs. Why *Allovahlkampfia*, *Naegleria* and *Didymium* twin-ribozyme introns generate full-length intron circles is not clear. One explanation could be that the full-length intron circles, which contain all the genetic information in the intron, are involved in intron mobility at the RNA level [5,13,18]. The generation of non-ligated SSU rRNA exons is likely to result in an imbalance in ribosomal components and generate nucleolar stress. Thus, intron circularization is expected to impact host physiology. Interestingly, all Heterolobosea amoeba studied to date have an unusual nucleolar structure consisting of several thousand identical copies of circular rDNA plasmids [39]. Loss of mature SSU rRNA due to full-length intron circularization could be compensated either by an increasing fraction of rDNA plasmids committed to transcription, or by increasing transcription initiation efficiency at each rDNA plasmid. These possibilities have to be experimentally investigated and evaluated.

### P2.1 is a conserved feature of lariat capping ribozymes

AspLC was found to be an efficient branching ribozyme *in vitro* with a number of conserved structural features in common with NaeLC (Figures 1A,B and 4A,B). Several compensatory changes in helices P3, P5, P6, P8, P9 and P15 were noted that further supported the general secondary structure diagram of the *Naegleria*-type LC ribozyme [17]. The most dramatic difference was found at the LC-GIR2 junction (boxed in Figures 1A,B and 2B), which contains almost no conserved nucleotides between AspLC and NaeLC. Here we propose that the LC-GIR2 junctions in *Allovahlkampfia* and *Naegleria* are involved in base-pairing interactions with the HE mRNA (Figure 2). This finding adds additional structural support to the observed mutual relationships between the LC ribozyme and the corresponding HE mRNA sequence in the *Naegleria*-type twin-ribozyme introns [15].

**Figure 5:**
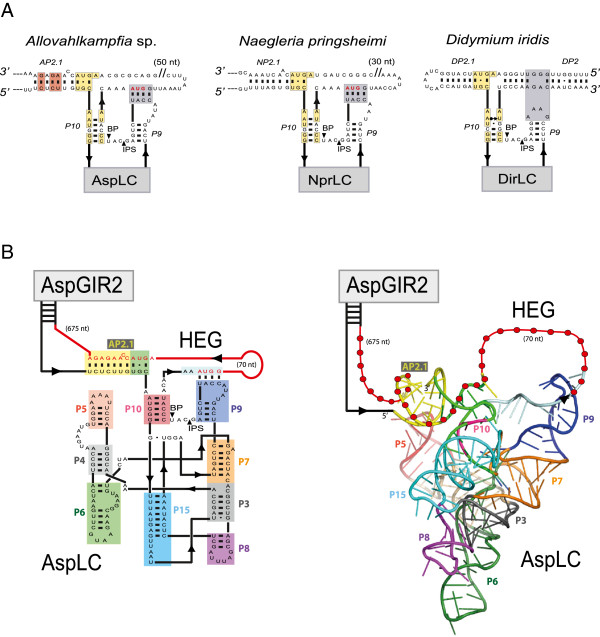
**P2.1 in LC ribozymes. (A)** Secondary structure diagrams of P2.1 in *Allovahlkampfia* (AP2.1), *Naegleria* (NP2.1) and *Didymium* (DP2.1) LC ribozymes. Yellow boxes: identical nucleotide positions in P2.1 and P10 of all three LC ribozyme variants. Red boxes: compensatory base-pair changes in P2.1 observed between the AspLC and NprLC ribozymes. Note that pHEG-1 in AspLC and NprLC has a similar location to that of the L9-tetraloop DP2 receptor interaction in DirLC [21], and that DP2.1 is a regular hairpin structure, while AP2.1/NP2.1 is made by a LC:HE mRNA interaction. **(B)** Structure model of the pre-cleaved AspLC ribozyme. Coloured sequence segments in the secondary structure (left) correspond to those in the 3D structure model (right). The HEG sequence (red line, dot) is involved in AP2.1 (yellow box).

The *Didymium* LC ribozyme contains an RNA structure element (DP2.1) shown to be essential in catalysis [40], but a corresponding P2.1 appears to be missing in the NaeLC ribozyme [10]. DP2.1 is supported by structural probing and mutational experiments [40], and the recent 2.5 Å DirLC crystal structure shows that DP2.1 bridges the catalytic core segments P10 and L5, thus participating in ribozyme activation [22]. Here we identified structures homologous to P2.1 in *Allovahlkampfia* (AP2.1) and *Naegleria* (NP2.1) LC ribozymes (Figure 5A). AP2.1 and NP2.1 are supported by sequence conservation to DP2.1, by the proximity to P10 and L9 interactions and by compensatory changes in the *Allovahlkampfia* and *Naegleria* helices (Figure 5A). The structural model of AspLC (Figure 5B) further suggests an integrated role of AP2.1, with a location that apparently corresponds to that of DP2.1 in the *Didymium* LC ribozyme [22]. We conclude that the peripheral helix P2.1 is a conserved feature of all LC ribozymes. P2.1 in *Allovahlkampfia* and *Naegleria* LC ribozymes appear unusual, owing to involvements of protein-coding sequences from the HEG region. However, it remains to be explored if AP2.1 and NP2.1 participate in conformational switch regulation of the branching reaction, and fold back onto the catalytic core, as seen for the DP2.1 [22,32].

## Conclusions

Our study expands the distribution pattern of nuclear twin-ribozyme introns among eukaryote microorganisms. Here we show that an intron in *Allovahlkampfia* sp. ribosomal DNA consists of a HEG and two ribozyme domains with distinct functions in splicing and mRNA maturation. One of the ribozymes is a typical group I intron ribozyme that catalyzes the intron splicing and intron circularization reactions. The other ribozyme represents a new member of the LC ribozyme class. The *Allovahlkampfia* LC ribozyme is responsible for forming a cap structure at the homing endonuclease mRNA, and thus contributes to intron mobility. Comparative analysis and 3D modelling support the hypothesis that mRNA coding sequences are directly involved in base parings with sequences of the LC ribozyme, forming a regulatory RNA element (P2.1) conserved among all member of the LC ribozyme class. This observation adds support to a mutual relationship between the LC ribozyme and corresponding homing endonuclease mRNA.

## Methods

### Templates and *in-vitro* transcription

The DNA fragment of Asp.S516 twin-ribozyme intron (lacking most (742 bp) of the HEG sequence) and parts of flanking exons were synthesized by Eurofins MWG Operon based on sequence information available in the accession number DQ388519 from the sequence database and cloned using the standard vector pEX-A (Eurofins MWG Operon). The authentic Asp.S516 sequence was confirmed by dideoxy sequencing analysis. Templates for *in-vitro* transcription were made by PCR using Pfu DNA polymerase. Primer sequences are compiled in Additional file 3: Table S1. All upstream primers contain the T7 polymerase promoter sequence. PCR products were purified with GeneJET PCR purification kit (Fermentas) and *in-vitro* transcribed by T7 RNA polymerase (Fermentas). Labelled transcript was transcribed using trace amount of [α-^32^P] uridine triphosphate, as previously described [41], and purified through Illustra MicroSpin S-200 HR columns. All transcripts were excised after gel electrophoresis on 5% denaturing (urea) polyacrylamide gels (UPAGs), eluted by diffusion into 0.25 M sodium acetate pH 6.0, 1 mM EDTA), followed by ethanol precipitation and resuspension in diethylpyrocarbonate-treated water.

### Cleavage analysis

Twin-ribozyme transcript was incubated at 45°C in splicing buffer (40 mM Tris-HCl pH 7.5, 25 mM MgCl_2_, 0.5 M KCl, 2 mM spermidine, 5 mM dithiothreitol) with or without 0.2 mM guanosine triphosphate. LC ribozyme RNA was pre-incubated at 45°C for 5 min in renaturation buffer (10 mM acetate buffer pH 5.5, 25 mM MgCl_2_, 1 M KCl) to promote correct folding. For kinetic cleavage experiments, the reactions were initiated by the addition of four volumes of start buffer (47.5 mM HEPES-KOH pH 7.5, 25 mM MgCl_2_, 1 M KCl) to the folded RNA. Aliquots of reaction were removed at various time points and terminated by addition of an equal volume of UBB (50% urea, 10 mM EDTA, 1 mg/ml bromophenol blue, 1 mg/ml xylene cyanol). RNA samples were run on 5% UPAG and exposed to storage phosphor screens. Interpretations of individual RNA species and processing reactions were made according to [42].

### Sequencing of circle junction and ligated exon junction

The twin-ribozyme intron transcript was incubated in splicing buffer for 5 min and 15 min with or without 0.2 mM guanosine triphosphate, respectively. Circularization RNA products were excised after gel electrophoresis on 5% UPAG, eluted by diffusion into 0.25 M sodium acetate pH 6.0, 1 mM EDTA, ethanol precipitated, and resuspended in diethylpyrocarbonate-treated water. Reverse transcription was performed using M-MuLV reverse transcriptase (Fermentas) and primer C734. cDNA was amplified by primers C734 and C735. The PCR product was then dideoxy sequenced with labelled primer C734 using a sequencing kit (USB) and separated on 8% UPAG. Ligated exon RNA was isolated after gel electrophoresis, subjected to reverse transcription using the primer C736, and amplified by PCR with C716 and C736. The PCR product was sequenced using labelled primer C736.

### ExoG-labelling

Unlabelled twin-ribozyme precursor transcript was incubated with [α-^32^P]guanosine triphosphate in splicing buffer at 45°C for 5 min. The reaction was terminated by adding equal amount of UBB and separated on 5% UPAG, together with kinetic cleavage reaction of labelled twin-ribozyme transcript.

### Data analysis of LC ribozyme cleavage reaction

RNA bands were quantified using ImageQuant 5.2 software and the kinetic data were fitted using one phase exponential decay with an endpoint correction:

$$\begin{array}{ll} {\left(\mathrm{Fraction}\;\mathrm{precursor}\right)}_{t}= & \;{\left(\mathrm{Fraction}\;\mathrm{precursor}\right)}_{t=\;\infty }\\ & +\;{\left(\mathrm{Fraction}\;\mathrm{precursor}\right)}_{t=\;0}\\ & \times \;\exp\left(‒k_{\mathrm{obs}}\;\times \;t\right) \end{array}$$

to obtain values for *k*_obs_ and endpoints of the reaction [41]. All cleavage reactions were performed three times in parallel and the results were highly reproducible.

### Primer extension

Primer extension analysis was used to map the 5′ end of the downstream LC RNA cleavage product (3′ RNA). The 3′ RNA was generated by self-cleavage reaction of the precursor LC RNA at various time points and annealed to end-labelled complementary primer C720. Reverse transcription was performed using M-MuLV reverse transcriptase (Fermentas), as previously described [40]. A dideoxy sequencing ladder was prepared from the corresponding PCR product using the same primer and run adjacent to the primer extension products as markers. The reactions were run on 8% UPAG.

### Molecular modelling

The AspLC ribozyme was built by homology modelling using Assemble software [43], taking the recent wild-type DirLC crystal structure (PDB ID 4p8z [22]) as a template. Differences in secondary structure and sequence were manually accommodated in the same software. One additional nucleotide was added in L8 and in J5/4. The extension of P6 was modelled as a loop E found between residues 216 and 235 of the *Deinococcus radiodurans* large ribosomal subunit in the 3PIP entry [44] of the pdb, except for the structures of L8 and J5/4. These were kept identical to DirLC, because the segments left unchanged are not expected to interfere with AP2.1. Figure 5B (right) was prepared using the PyMOL program [45].

## Abbreviations

AspLC: *Allovahlkampfia* LC ribozyme; DirLC: *Didymium* LC ribozyme; EDTA: ethylenediaminetetraacetic acid; exoG: exogenous guanosine cofactor; HE: homing endonuclease; HEG: homing endonuclease gene; IPS: internal processing site; LC: lariat capping; LSU: large subunit; NCBI: National Center for Biotechnology Information; NaeLC: *Naegleria* LC ribozyme; NprLC: *N. pringsheimi* LC ribozyme; PCR: polymerase chain reaction; RT-PCR: reverse transcription polymerase chain reaction; SS: splice site; SSU: small subunit; UBB: 50% urea, 10 mM EDTA, 1 mg/ml bromophenol blue, 1 mg/ml xylene cyanol; UPAG: urea polyacrylamide gel.

## Competing interests

The authors declare that they have no competing interests.

## Authors’ contributions

YT performed the experimental work and participated in analyses and discussions of the results. BM performed 3D RNA modelling and participated in discussions of the results. PPG participated in bioinformatic discovery and discussion of the results. SDJ and HN directed the research. SDJ, HN and YT wrote the manuscript in collaboration with all authors. All authors read and approved the final manuscript.

## Supplementary Material

Additional file 1: Figure S1 Unrooted neighbour-joining analysis based on twin-ribozyme intron sequences from Naegleria and Allovahlkampfia isolates. Click here for file

Additional file 2: Figure S2 Amino acid alignment of nine Naegleria homing endonuclease sequences and that from Allovahlkampfia. Click here for file

Additional file 3: Table S1. Key features of primer sequences used in this study. Click here for file
