# Supplementary material for: Molecular characterization of a new member of the lariat capping twin-ribozyme introns
Source: Mob DNA. 2014 Sep 15;5:25. doi: 10.1186/1759-8753-5-25 (PMC4167309; doi:10.1186/1759-8753-5-25)

Additional file

Additional file 1: Figure S1. Neighbor-Joining (NJ) analysis (unrooted) based on twin-ribozyme intron sequences, including HEGs from nine representative *Naegleria* isolates and the *Allovahlkampfia* sp. NJ bootstrap values (500 replicates) above 50% are shown at the branches. The selected twin-ribozyme intron sequences were aligned by ClustalW and the phylogenetic tree was constructed with NJ method using MEGA version 4.0. Analyzed sequences include *N. jamiesoni* (X78279); *N. pringsheimi* (AM167882); *N. andersoni* (Z16417); *N. italica* (X78277); *N. philippinensis* (AM167881); *N. carteri* (AM167878); *N. clarki* (X78281); *Naegleria* sp. NG358 (AM167883); *Naegleria* sp. NG393 (AM167884); *Allovahlkampfia* sp. (DQ388519).

Additional file 1: Figure S1

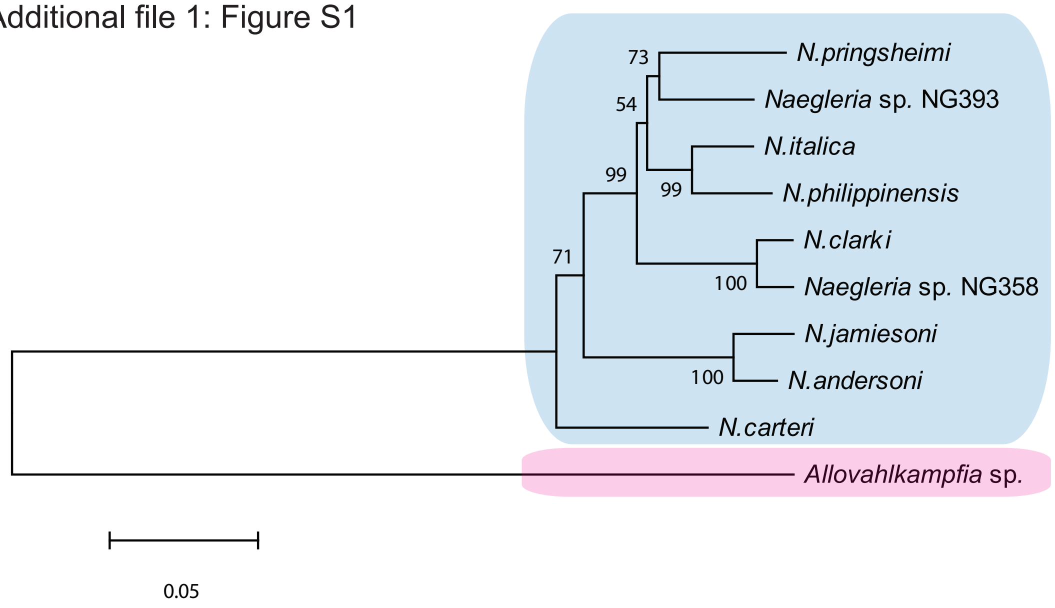

Supplement: Additional file 1: Figure S1 — Unrooted neighbour-joining analysis based on twin-ribozyme intron sequences from Naegleria and Allovahlkampfia isolates. [file 1759-8753-5-25-S1.pdf]
