# Supplementary material for: Molecular characterization of a new member of the lariat capping twin-ribozyme introns
Source: Mob DNA. 2014 Sep 15;5:25. doi: 10.1186/1759-8753-5-25 (PMC4167309; doi:10.1186/1759-8753-5-25)
Supplement: Additional file 2: Figure S2 — Amino acid alignment of nine Naegleria homing endonuclease sequences and that from Allovahlkampfia. [file 1759-8753-5-25-S2.pdf]

Additional file

Addtional file 2: Figure S2. Amino acid alignment of nine selected *Naegleria* homing endonuclease sequences and that from *Allovahlkampfia*. Identical amino acid positions to I-*Nja*I are indicated by dots and deletions by dashes. I-*Nja*I from *N. jamiesoni* (X78279); I-*Npr*I from *N. pringsheimi*; I-*Nan*I from *N. andersoni*; I-*Nit*I from *N. italica*; I-*Nph*I from *N. philippinensis*; I-*Nca*I from *N. carteri*; I-*Ncl*I from *N. clarki*; I-NG358 from *Naegleria* sp. NG358; I-NG393 from *Naegleria* sp. NG393; I-*Asp*I from *Allovahlkampfia* sp. Functional important residues involved in zinc binding (C and H) and catalysis (N) are boxed.

Additional file 2: Figure S2

```
I-NjaI:  MV-----SIKQKVTRLRNKLRSRSTRNGPKTITVKIDRKSFKNGYDPLVDTIDY--GYSKMAKITVNKNDQLAKLKNCKQAVNIFNEWLS
I-NprI:  ..-----T...M.RT....V.AS..TKR.........LE..........KN...--..........PG..R.S..G.R.T.R.V..D...
I-NanI:  ..-----..............A...LS.H....................N....--.......................K..........
I-NitI:  ..-----T.RKM.R...KR..I...TKR..........E..........KS...--..........A.C.R.S..N.R...K........
I-NphI:  ..-----..TKM.RS..KR..V...TKR.........LER.........K....--..........A.C.R.S..G.R...R.......L
I-NcaI:  ..-----...E..KK..KR.LTG..SRY..........E...D......NI...--..........A.K.R.S..DSR...K.......T
I-NclI:  ..-----T.RKI.K.......A...TKR.I........E..........K....--..N...R..LQ.S.R.S..D.R...K........
I-NG358: ..-----T.RKI.K.......A...TKR.R........E..........NS...--..N...R..LQ.S.R.S..D.R...K........
I-NG393: ..-----T..KM.K.......A...TKH..I..................N....--..........TQS.R.S..S.R.M.R........
I-AspI:  ..KYFPYGHIIHRLGR..I.LG.ASTKR-..KK.I..ASQ.RD.F.S.IE.NYHKK..E.L.NAPNV.E-P.G.GNALSAGKSKSYFHRW

I-NjaI:  NRKGDK--GRSGKQKPYCFDELKKLDVCKHEFGECLIGAASKTKSGFKVRFMNNKGSDSYVHHVSVFAKSTCENCIHSRKMLETVSSSKK
I-NprI:  T...E.--........F.....E.................T............K...........A..N..N.D...VE...........
I-NanI:  ......--................................N............D...........A..N.....................
I-NitI:  .H....--....................E...........N..........L.............A..N..N.....VE...........
I-NphI:  ......--....................E...........N..........L.............A..N..N....TVE...........
I-NcaI:  ....E.--........F.........I.............N........................A..N..NV....LE...........
I-NclI:  .C....--........FV........I.N...........N...A......L.KQ..........A..N..A.....VE...........
I-NG358: ......--........FV........I.N...........N...A......L.KQ..........A..N..T.....IE...........
I-NG393: ......--........F.........I.E...........N..........L.KQ..........A..N..T.....VE.......R...
I-AspI:  ANNRSRGT..M.R.SRTYIPT.T.VLC.E.G.....L..SN.S.....CS.LSD....G......IL.N.RSSDP..KLALIKK..QR..

I-NjaI:  DPDARTISHLCGNGGCARPGHLRIEKKSVNDERTHCHFLLRRSQSVAQSEMIRLACPHTPRCFVNLYKINKPYY
I-NprI:  .SE.............S............................LH.............K....I........
I-NanI:  ...........................T.................L...................I........
I-NitI:  ..E..........................................RN..........S.K....M....T...
I-NphI:  .T...........................................RN..........S.K....M....T...
I-NcaI:  .D..............................C............LH......M...........I........
I-NclI:  .NE..........................................IN.............K....I....T...
I-NG358: .SE..........................................IN.............K....I....T...
I-NG393: EN..............................N............RH..........S......I....T...
I-AspI:  EKN.YSV..............II..P.T.....VA..RF..SCKCKWDARV..RL...K.K....T.VGI.A..
```
