# Supplementary material for: Molecular characterization of a new member of the lariat capping twin-ribozyme introns
Source: Mob DNA. 2014 Sep 15;5:25. doi: 10.1186/1759-8753-5-25 (PMC4167309; doi:10.1186/1759-8753-5-25)
Supplement: Additional file 3: Table S1. — Key features of primer sequences used in this study. [file 1759-8753-5-25-S3.pdf]

Additional file 3: Table S1. Primer sequences

Primer	Sequence (5´→3´)
C716	AAT TTA ATA CGA CTC ACT ATA GGT TAG ATT AAA ATT CTA TTA G
C717	TAT TAA TAT ACG CTA CTA GAG C
C718	AAT TTA ATA CGA CTC ACT ATA GGC GAC TTT CTT TTT CTC TTG
C719	GAA ATA TTT AAC CAT TTT GTA TG
C720	GAA ATA TTT AAC CAT
C734	CTA TCA CTG TAT CAA CAG TGG
C735	TCC AAT GGA AAA GGA TGA GC
C736	GCC CTC TAG ATG CAT GCT CG
